# Étude rétrospective concernant la prise en charge chirurgicale des lombosciatiques communes dans le Département d'Orthopédie de Tataouine, Tunisie: à propos de 44 cas

**DOI:** 10.11604/pamj.2020.35.103.22510

**Published:** 2020-04-07

**Authors:** Mourad Hammami, Nizar Sahnoun

**Affiliations:** 1 Service de Chirurgie Orthopédique et Traumatologie, Hôpital Régional de Tataouine, Tataouine, Tunisie; 2 Service de Chirurgie Orthopédique et Traumatologie, CHU Habib Bourguiba, Sfax, Tunisie

**Keywords:** Lombosciatiques communes, chirurgie, indication, Lumbosciatica, surgical, indication

## Abstract

La lombosciatique commune représente un problème de santé publique par son retentissement socioprofessionnelle. Le but de notre travail est d'étudier l'indication du traitement chirurgical et la place de chaque technique utilisé. Il s'agit d'une étude rétrospective réalisée au Service d'Orthopédie de Tataouine, concernant 44 patients présentant une lombosciatique commune et ayant eu un traitement chirurgical durant la période allant de 2013 à 2018. La fiche de renseignement a inclus les données épidémiologiques et les données cliniques. Les patients ont eu un bilan radiologique comportant une radiographie du rachis lombaire face et profil et une tomodensitométrie (TDM) lombaire qui a précisé l'étiologie de la sciatalgie commune. Le traitement chirurgical a été indiqué après échec d'un traitement médical, en cas de sciatique hyperalgique et en cas de complication neurologique. Dans notre étude, la hernie discale était la principale étiologie de lombosciatique (50% des cas) suivie par le canal lombaire étroit (25%), le spondylolisthésis (22,7%) et la méga apophyse transverse de L5 (2,3%). La dissectomie classique était la technique utilisée pour la cure chirurgicale des hernies discales. Huit patients présentant un spondylolisthésis ont bénéficié d'une laminectomie associée à une arthrodèse postérieure. Pour la cure chirurgicale de canal lombaire rétréci, on a pratiqué une laminectomie seule dans 54,54% des cas. L'évolution au recul a été favorable dans 90% des cas l'échelle visuelle analogique (3±1 au recul). Le choix des techniques chirurgicaux dépend de l'étiologie et des données de l'imagerie qui imposent le choix des étages de l'arthrodèse.

## Abstract (en)

Lumbosciatica is a public health problem because of its socio-professional impact. The purpose of our study is to evaluate the indication for surgical treatment and the role of each technique used. We conducted a retrospective study in the Department of Orthopaedics in Tataouine. The study involved 44 patients with common lumbosciatica and having undergone surgical treatment over the period from 2013 to 2018. The information sheet included the epidemiological data and the clinical data. The patients underwent radiological assessment including lumbar spine x-ray (frontal and lateral views) and lumbar computed scan (CT) scan which clarified the cause of sciatica. Surgical treatment was indicated after medical treatment failure, in patients with hyperalgesia and in patients with neurological complication. In our study, herniated disc was the primary cause of lumbosciatica (50% of cases) followed by lumbar spinal stenosis (25%), spondylolisthesis (22.7%) and transverse mega-apophysis of L5 vertebrae (2.3%). Traditional discectomy was the most used technique for surgical treatment of herniated disc. Eight patients had spondylolisthesis. They underwent laminectomy associated with posterior arthrodesis. Lumbar spinal stenosis was treated with laminectomy alone in 54.54% of cases. Outcome was favorable during the follow up period in 90% of cases (Visual Analog Scale 3±1 at follow-up). Surgical technique depends on etiology and imaging data on which to base the choice of arthrodesis stages.

## Introduction

La lombosciatique commune non compliquée est une affection d'évolution spontanément favorable dans la majorité des cas: 80% des patients guérissent dans un délai de 8 semaines et 95% des patients guérissent sans intervention chirurgicale dans un délai d'un an [1]. Les étiologies de ces lombosciatiques sont dominées par la hernie discale [2]. L'efficacité du traitement chirurgical est bien démontrée avec un délai de guérison amélioré d'environ 50% par rapport au traitement médical [1]. A travers notre série, nous avons évalué nos résultats tout en se comparant aux données de la littérature.

## Méthodes

**Figure 1:**
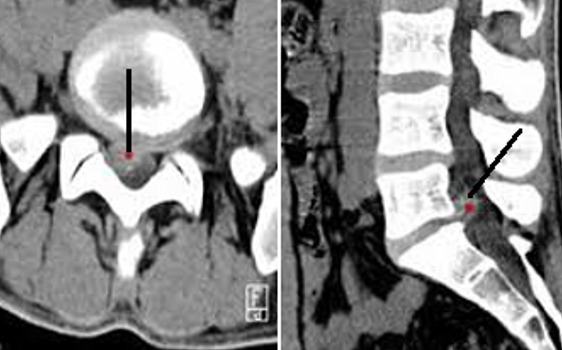
TDM en coupe axiale et sagittale montrant une hernie discale L5-S1

**Figure 2:**
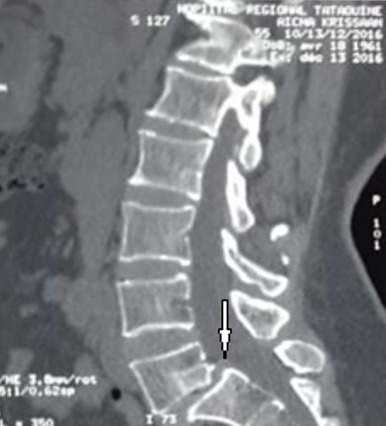
TDM du rachis lombaire en coupe en coupe sagittale montrant un SPL L5-S1 grade 1

**Figure 3:**
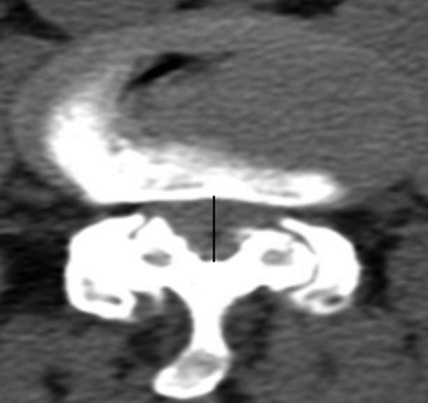
TDM en coupe axiale montrant une sténose canalaire centrale

**Figure 4:**
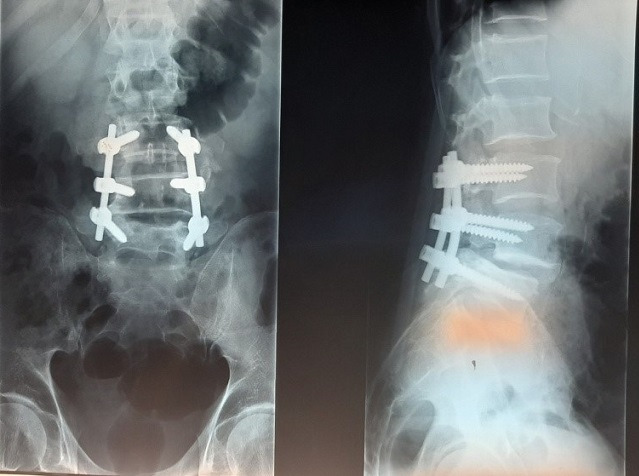
Traitement d'une SPL L5-S1 stade 1 par laminectomie et arthrodèse L4-S1

**Figure 5:**
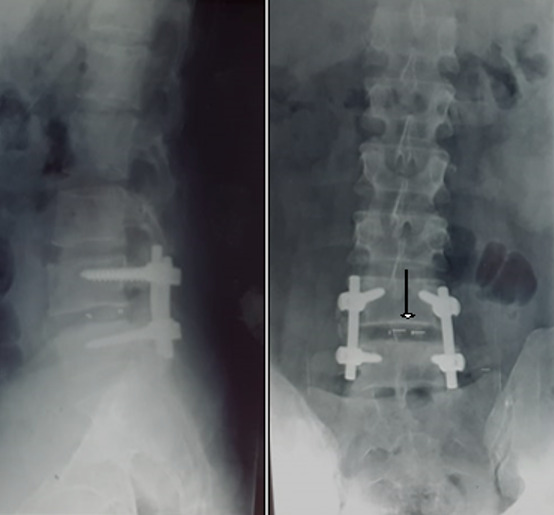
Un cas de SPL L4 L5 grade I chez un patient de 53 ans traité par la technique de PLIF

Il s'agit d'une étude descriptive rétrospective réalisée au Service d'Orthopédie de Tataouine, concernant 44 patients présentant une lombosciatique commune et ayant été traité chirurgicalement durant la période allant de janvier 2013 à décembre 2019. On a établi une fiche de renseignements comportant les données épidémiologiques à savoir l'âge, le sexe, la profession et les données cliniques, le type de douleur, son siège, son irradiation, le mode d'évolution et son début, tous nos patients ont eu un bilan radiologique comportant une radiographie du rachis lombaire face et profil et une TDM lombaire qui a précisé l'étiologie de la sciatalgie commune (Figure 1). Le traitement chirurgical a été indiqué après échec d'un traitement médical, en cas de sciatique hyperalgique et en cas de de complication neurologique. En cas d'hernie discale, une dissectomie classique associée à une libération des racines nerveuses comprimées a été réalisé par un petit abord postérieur unilatéral du côté de la hernie. Une laminectomie partielle est parfois nécessaire, le sac dural est refoulé vers la ligne médiane ainsi que l'épaule de la racine pour permettre l'abord de la hernie. La hernie est enlevée après incision du ligament vertébral postérieur si elle n'est pas exclue. La dissectomie se poursuit dans l'espace discal jusqu'à ce qu'elle ne rapporte plus de fragments discaux. La liberté de la racine est vérifiée à l'aide d'un palpeur jusque dans le foramen. Pour le spondylolisthésis (SPL), on a réalisé une laminectomie associée à une arthrodèse postérieure ou arthrodèse inter somatique lombaire par voie post posterior lumbar inter-body fusion (PLIF) (Figure 2, Figure 3, Figure 4, Figure 5). Pour le canal lombaire rétréci, on a pratiqué une laminectomie associée ou non à une arthrodèse et si besoin un recalibrage du canal a été associé au geste. La satisfaction des patients en postopératoire est évaluée à l'aide des scores d'auto-évaluation: l'échelle visuelle analogique (EVA). L'ensemble des données ont été saisies et analysées au moyen de logiciel SPSS. Les variables qualitatives ont été décrites par les moyennes alors que les variables quantitatives ont été décrites par les effectifs et les pourcentages.

## Résultats

L'âge moyen de notre population est 51 ans avec une légère prédominance masculine (24 hommes et 20 femmes). La lombosciatique résistante au traitement médical a été notée dans 36 cas, la sciatique paralysante dans 7 cas et une sciatique compliqué de syndrome de la queue de cheval dans un cas. La hernie discale était la cause dans 22 cas, un canal lombaire rétréci dans 11 cas et le spondylolisthésis a été trouvée dans 10 cas, on a noté un cas de méga-apophyse transverse. Huit patients ont bénéficié d'une laminectomie associée à une arthrodèse postérieure classique étendue sur un étage (4 cas), 2 étages (3 cas) ou 3 étages (1 cas) et deux patients ont bénéficié d'une arthrodèse inter somatique lombaire par voie post PLIF. Pour la cure chirurgicale de canal lombaire rétréci et les cas de sondylolistésis (Figure 1), on a pratiqué une laminectomie seule étendue sur un étage (2 cas) ou 2 étages (4 cas), une laminectomie associée à une arthrodèse étendue sur un étage (1 cas), 2 étages (2 cas) ou 3 étages (1 cas) et un recalibrage associé à une arthrodèse étendue sur 2 étages dans 1 cas. Une résection de méga apophyse transverse droite de L5 chez un patient. Une brèche iatrogène de la dure-mère a été constatée chez 5 patients. La douleur sciatique a disparu dans 90% des cas (3±1 au recul) le reste des patients gardent des lombalgies modérés. La persistance des troubles neurologiques a été observée chez 3 patients, soit 6,8% des cas.

## Discussion

Dans notre série la hernie discale était la cause de lombosciatique dans 50% des cas, le canal lombaire étroit (CLE) dans 25% des cas, le spondylolisthésis (SPL) dans 22,7% des cas et la méga apophyse transverse dans 2,3% des cas ce qui rejoint bien la littérature [2, 3], la lombosciatique discale est trouvé dans la moitié des cas, la SPL dans 12,9% des cas et le CLE dans 11,1% des cas. Le traitement chirurgical est de plus en plus indiqué pour les sciatalgies, aux Etats Unis 240 000 interventions sont pratiquées par an [4]. Dans notre série 81,1% des patients ont été opérés pour une lombosciatique hyperalgique, définit par l'OMS comme toute douleur ressentie insupportable et résistante aux antalgiques majeurs de palier 3, et 15,9% pour des lombosciatiques paralysante définit comme un déficit moteur d'emblée inférieur à 3 selon l'échelle MRC [5]. La dissectomie reste le traitement chirurgical habituel de la cure de la hernie discale [6]. Les techniques chirurgicales utilisées pour le traitement de spondylolisthésis sont nombreuses et diffèrent selon les habitudes de chaque chirurgien [6]: soit arthrodèse inter somatique lombaire par voie postérieure: posterior lumbar inter body fusion (PLIF) [7], soit voie latérale trans foraminale: arthrodèse inter somatique lombaire par voie trans foraminale: trans foraminal lumbar inter body fusion (TLIF), soit un abord antérieur: arthrodèse inter somatique par voie antérieure: anterior lumbar inter body fusion (ALIF) [8], soit une libération avec arthrodèse postérieure classique. Dans notre étude, l'arthrodèse postérieure classique était la technique la plus utilisée (80% des cas). La décompression large pour sténose dégénérative globale se fait soit par une décompression interne économique pour sténose dégénérative globale appelée encore recalibrage ou fenestration. Ces 2 techniques peuvent être associées à une stabilisation surtout en présence d'un listhésis dégénératif [9]. Pour la cure chirurgicale d'une méga apophyse transverse de L5, son traitement repose sur la résection de la méga apophyse de L5 surtout dans les cas rebelles au traitement médical [10]. Les complications per opératoires: les brèches dure-mériennes [11] constatées dans 11,4% des cas dans notre série, une lésion radiculaire [12] et des complications vasculaires: les plus rares mais les plus graves [13]. Ces deux dernières complications n'ont pas été retrouvées dans notre série. Nous n'avons pas eu de sepsis dans notre série, bien que dans la littérature l'infection est la complication la plus fréquente en postopératoire [14]. Still *et al*. [14] et Saleh *et al*. [15] rapportent respectivement un pourcentage d'infection postopératoire de 1,71% et 1,03%, soit les complications neurologiques qui sont en général secondaires soit à un problème technique peropératoire, soit à un hématome épidural postopératoire, soit à une compression résiduelle. La satisfaction des patients reste l'objectif majeur, qui peut être évaluée à l'aide de l'échelle visuelle analogique (EVA) [16] comme c'est le cas de notre travail bien que le score d'Oswestry (*Oswestry disability index*) reste un moyen d'évaluation plus objectif.

## Conclusion

La lombosciatique commune est une affection très fréquente dont l'évolution est favorable dont la grande majorité des cas. Le traitement chirurgical est indiqué en cas de lombosciatique hyperalgique rebelle au traitement médical, de lombosciatique paralysante et en cas de syndrome de queue de cheval. Le choix des techniques chirurgicaux dépend de l'étiologie et des données de l'imagerie qui imposent le choix des étages de l'arthrodèse.

### Etat des connaissances actuelles sur le sujet

- Les lombosciatiques communes sont un motif de consultation fréquent;
- Les lombosciatiques communes posent un problème de santé publique;
- Le traitement initial des lombosciatiques communes est essentiellement médical.

### Contribution de notre étude à la connaissance

- Le traitement chirurgical trouve de plus en plus sa place dans le traitement de la lombosciatique;
- L’indication bien adaptée à chaque étiologie permet d’avoir de bons résultats fonctionnels;
- Indiqué à temps, le traitement chirurgical donne de bons résultats fonctionnels.

## Conflits d'intérêts

Les auteurs ne déclarent aucun conflit d'intérêts.

## References

[1] Legrand E, Bouvard B, Audran M, Fournier D, Valat JP, Section Rachis de la SFR (2007). La sciatique par hernie discale: traitement conservateur ou traitement radical. Revue du Rhumatisme.

[2] Bejia I, Younes M, Zrour S, Touzi M, Bergaoui N (2004). Les facteurs associés à l’évolution de la sciatique commune: à propos de 1092 cas. Revue du Rhumatisme.

[3] Mostofi K, Peyravi M, Moghaddam BG (2019). A comparison of sciatica in young subjects and elderly person. Journal of Clinical Orthopaedics and Trauma.

[4] Deyo RA (2007). Back Surgery-Who Needs It?. N Engl J Med.

[5] Selz Amaudruz Florence, Morard Marc, Buchard Pierre-Alain, Frochaux Vincent (2010). When should a patient with low back pain/sciatica be referred to the emergency ward. Revue médicale suisse.

[6] Sailhan F (2014). Les techniques chirurgicales utilisées dans les pathologies discales lombaires dégénératives. La Lettre du Rhumatologue.

[7] Steffee AD, Sitkowski DJ (1988). Posteriorlumbarinterbody fusion and plates. Clin Orthop Relat Res.

[8] Capener N (1932). Spondylolisthesis. Br J Surg.

[9] Katz JN, Harris MB (2008). Lumbar Spinal Stenosis. N Engl J Med.

[10] Ugokwe KT, Chen T-L, Klineberg E, Steinmetz MP (2008). Minimally Invasive SurgicalTreatment of Bertolotti’s Syndrome: Case Report. Operative Neurosurgery.

[11] Ahn Y, Lee HY, Lee S-H, Lee JH (2011). Dural tears in percutaneous endoscopic lumbardiscectomy. EurSpine J.

[12] Airaksinen O, Herno A, Turunen V, Saari T, Suomlainen O (1997). Surgical outcome of 438 patients treated surgically for lumbar spinal stenosis. Spine.

[13] Lacombe M (2006). Les complications vasculaires de la chirurgie discale lombaire. Annales de Chirurgie.

[14] Still MEH, Venturini S, Vycheth I, Nang S, Vuthy D, Park KB (2019). Predictive Factors of Spine Surgery Complications at a Major Government Hospital in Cambodia. World Neurosurgery.

[15] Saleh A, Thirukumaran C, Mesfin A, Molinari RW (2017). Complications and readmission after lumbar spine surgery in elderly patients: an analysis of 2,320 patients. The Spine Journal.

[16] Chapin L, Ward K, Ryken T (2017). Preoperative Depression, Smoking, and Employment Status are Significant Factors in Patient Satisfaction After Lumbar Spine Surgery. Clin Spine Surgery.

